# Blood Product Transfusion Patterns in One Lithuanian and Two Polish Academic Emergency Departments: A Retrospective Comparative Study

**DOI:** 10.3390/medicina62101918

**Published:** 2026-10-05

**Authors:** Gailė Damulevičiūtė, Kristina Blažienė, Jakub Nożewski, Greta Miškinaitė, Goda Monstavičiūė, Kinga Janiszewska, Tomasz Kłosiewicz, Dawid Woszczyk, Patryk Konieczka, Diana Remeikienė, Vytautas Aukštakalnis

**Affiliations:** 1 Emergency Medicine Department, Lithuanian University of Health Sciences, 44307 Kaunas, Lithuania; 2 Emergency Department, Collegium Medicum, Nicolaus Copernicus University in Toruń, Jagiellońska 13-15, 85-067 Bydgoszcz, Poland; jakub.nozewski@biziel.pl; 3 Lithuanian University of Health Sciences, 44307 Kaunas, Lithuania; 4 Department of General and Minimally Invasive Surgery, Jan Biziel University Hospital No. 2, 85-168 Bydgoszcz, Poland; 5 Department of Medical Rescue, Poznan University of Medical Sciences, 7 Rokietnicka Street, 60-806 Poznań, Poland; 6 Doctoral School, Poznan University of Medical Sciences, 10 Fredry Street, 61-701 Poznań, Poland; d.woszczyk99@gmail.com; 7 Department of Emergency Medicine, Poznan University of Medical Sciences, 7 Rokietnicka Street, 60-806 Poznań, Poland; 8 Department of Oncology and Hematology, Institute of Oncology, Lithuanian University of Health Sciences, Eivenių Str. 2, 50009 Kaunas, Lithuania

**Keywords:** blood transfusion, blood product management, emergency department, patient blood management, restrictive transfusion strategy

## Abstract

*Background and Objectives*: Blood product transfusion is an important emergency intervention for patients with acute bleeding, severe symptomatic anemia, and selected hematological conditions. Patterns of ED transfusion may differ according to the local case mix, referral pathways, and blood bank processes. We compared transfusion-related characteristics and outcomes among patients treated in one Lithuanian and two Polish academic emergency departments. *Materials and Methods*: We performed a retrospective comparative study of adults (aged ≥18 years) in whom a blood product transfusion was initiated in the ED between 1 January 2022 and 31 December 2024 at one Lithuanian and two Polish academic hospitals. We compared patient characteristics, indication categories, shock index values, time to transfusion, blood product use, adjunctive therapies, laboratory values, ED disposition, hospital length of stay, and in-hospital mortality. Findings are reported as center-cohort comparisons and are not intended to represent national practice. *Results*: A total of 670 patients were included: 367 at the Lithuanian center and 303 across the two Polish centers. GI bleeding was significantly more common in the Lithuanian center cohort (48.8% vs. 22.8%; OR 3.22 [95% CI 2.30–4.53]; *p* < 0.001), whereas anemia and malignancy-related anemia were more frequent in the Polish center cohort (anemia: 34.7% vs. 18.3%, OR 0.42 [95% CI 0.29–0.6]; *p* < 0.001; cancer: 26.4% vs. 7.6%, OR 0.23 [95% CI 0.15–0.37]; *p* < 0.001). The median shock index was significantly higher in the Lithuanian center cohort than in the Polish center cohort (0.9; [IQR 0.7–1.2] vs. 0.7; IQR 0.6–0.9; 0.2 [95% CI 0.1–0.3]; *p* < 0.001), and pre-transfusion hemoglobin was lower (64.5 g/L [IQR 50–86] vs. 119 g/L [IQR 83–139]; *p* < 0.001). Uncrossmatched RBC units were more frequently administered in the Lithuanian center cohort (133 (36.9%) vs. 47 (15.6%); OR 3.18 [95% CI 2.18–4.64]; *p* < 0.001), as was fresh frozen plasma (71 (19.5%) vs. 3 (1%); OR 24.23 [95% CI 7.55–77.8]; *p* < 0.001). Conversely, platelet transfusions were more common in the Polish center cohort (17 (4.6%) vs. 45 (14.9%), OR 0.28 [95% CI 0.16–0.5]; *p* < 0.001). The median time from ED arrival to transfusion was longer in the Lithuanian center cohort than in the Polish center cohort (150 [IQR 75–256] vs. 117 [IQR 103–154], 23 [95% CI 9–39]; *p* = 0.002). Hospital admission was significantly more frequent in the Lithuanian cohort than in the Polish cohort (350 (95.4%) vs. 112 (37.0%); *p* < 0.001). Among admitted patients, the median hospital length of stay was significantly longer in the Lithuanian cohort compared with the Polish cohort (6 days [IQR 3–11] vs. 1 day [IQR 1–5]; 95% CI for the difference 3–4; *p* < 0.001). In-hospital mortality was 33.5% (*n* = 123) in the Lithuanian cohort and 13.9% (*n* = 42) in the Polish cohort (OR 3.13 [95% CI 2.12–4.63]; *p* < 0.001). *Conclusions*: Among transfused patients in the three participating academic EDs, the case mix, blood product use, transfusion timing, and unadjusted outcomes differed substantially. These center-specific findings may inform future prospective studies of how referral pathways, clinical populations, and local transfusion processes influence ED blood product utilization. They do not establish differences in national practice, transfusion appropriateness, or causal effects on clinical outcomes.

## 1. Introduction

Approximately 120.4 million blood donations are collected annually worldwide, underscoring the essential role of blood transfusion services in modern healthcare systems [1]. Blood transfusion remains one of the most frequently performed potential life-saving interventions and represents a critical contribution to emergency, trauma, surgical, and critical care medicine [2,3,4,5,6,7,8].

In the emergency department (ED), blood transfusions are a vital therapeutic intervention for patients with acute hemorrhage, severe symptomatic anemia, coagulopathy, and other immediately life-threatening conditions requiring rapid hemodynamic stabilization [2,3,4,5,6]. The contribution of emergency medicine to overall hospital blood product utilization is substantial. A four-year French tertiary hospital retrospective study reported a median Yearly Adjusted Number of Transfusions per ED Patient (YANTED) of 16.3 red blood cell (RBC) units per 1000 adjusted ED patients, while ED transfusions accounted for 11% of all hospital RBC transfusions [9].

Although only a small proportion of ED patients require transfusion, they frequently represent a high-acuity population with substantial morbidity and mortality risk. National blood donation data provide context for the importance of appropriate blood product stewardship and reliable product availability. The Lithuanian Institute of Hygiene reported 60,345 blood donors and 117,663 donations in Lithuania in 2024, whereas Statistics Poland reported 651,400 donors and 1,548,400 blood and blood component donations in Poland in the same year [10,11]. Transfusion practice therefore remains an important target for quality improvement initiatives and patient blood management (PBM) programs [2,7,8].

A restrictive RBC transfusion strategy (hemoglobin (Hb) threshold < 70–80 g/L) is recommended over a liberal strategy (Hb < 90–100 g/L) for most hemodynamically stable and critically ill patients; however, these restrictive thresholds are not applicable for all clinical cohorts, and guidelines distinguish high-risk presentations such as acute coronary syndrome, uncontrolled hemorrhage or trauma [2,4,5,6,8]. Blood component transfusions in the ED are administered for bleeding (both traumatic and non-traumatic) [12,13,14,15,16,17], symptomatic anemia [12,13,16], and chronic anemia associated with malignant disease [12,15,16,17,18,19], hemato-oncological diseases [12,15,18,19] or other chronic diseases [12,15,17,18,19]. Plasma and platelet transfusions may also be administered in patients with clinically significant coagulopathy or thrombocytopenia [12,13].

Gastrointestinal (GI) bleeding appears to be one of the most common indications for blood transfusion in the emergency department [13,14,15,20,21]. However, other studies have identified different leading indications, including symptomatic or severe anemia [12,16] and chronic anemia associated with onco-hematological malignancies [18]. In contrast, transfusions related to traumatic injuries in the emergency department have predominantly been investigated in dedicated trauma-focused studies [22,23,24,25,26,27,28,29,30].

Packed RBCs represent the most frequently utilized blood component in EDs, accounting for approximately 52–65% of all requested blood products [14,31]. Platelet concentrates and fresh frozen plasma constitute the next most frequently utilized components, while coagulation factor concentrates are increasingly incorporated into modern hemostatic management strategies [14,31]. According to Özdamar et al., “the main indications for requesting blood products were gastrointestinal bleeding and anemia. Specifically, 30.04% of erythrocyte suspensions, 31.9% of thrombocyte suspensions, and 60.56% of fresh frozen plasma were used for patients with gastrointestinal bleeding. In trauma patients, 12.75% of requested erythrocyte suspensions, 0.083% of thrombocyte suspensions, and 13.89% of fresh frozen plasma were utilized’’ [13]. The World Health Organization’s 2024 implementation guidance defines blood as an organ and mandates PBM integration across emergency admissions to curb worldwide healthcare expenditure and avoid unnecessary transfusions [32].

Differences in ED transfusion patterns can arise from the local case mix, referral pathways, the availability of outpatient transfusion services, blood bank logistics, emergency-release product policies, and admission practices. Cross-center data describing how these factors are reflected in ED transfusion populations remain limited, particularly in Central and Eastern Europe. The participating academic centers in Lithuania and Poland provide an opportunity to compare transfused ED populations within different local organizational settings. This study was designed as a retrospective descriptive comparison of transfusion-related characteristics in one Lithuanian and two Polish academic EDs, rather than as an assessment of national transfusion practice, transfusion appropriateness, or adherence to PBM recommendations.

The primary objective was to compare patient characteristics, clinical indication categories, shock index values, blood product use, time from ED arrival to transfusion initiation, adjunctive therapies, selected laboratory values, ED disposition, hospital length of stay, and in-hospital mortality among patients transfused in the participating EDs. We hypothesized that the transfused populations and documented product-use patterns would differ between the participating center cohorts. We did not assess clinician-level transfusion rationale, protocol adherence, or causal effects of transfusion practice on outcomes.

## 2. Materials and Methods

We conducted a retrospective, multicenter, comparative, descriptive study following the STROBE guidelines for observational studies. This study compared blood transfusion practices across three emergency departments located in two Central European countries, the Hospital of the Lithuanian University of Health Sciences (Lithuania), Jan Biziel University Hospital (Poland) and Poznan University of Medical Sciences (Poland), from 1 January 2022 to 31 December 2024. The design was explicitly comparative, with the primary aim of identifying similarities and differences in transfusion decision-making frameworks rather than establishing causal relationships or determining the superiority of one approach over another.

While institutional protocols for blood product ordering and transfusion authorization are present at each site, they were not systematically compared as part of this study. Each institution confirmed that transfusion decisions are made at the bedside by attending physicians in consultation with blood bank staff, with no mandatory computerized decision support at the time of this study.

Data were extracted from electronic medical records using standardized case report forms by trained research coordinators at each institution. Patients were identified through blood bank transfusion records (the authoritative source for patient identification) and cross-referenced with ED admission records.

Inclusion criteria: all adult (≥18 years old) patients presenting to the ED for any reason with blood transfusion initiated in the emergency department.

Analyzed variables: age, gender, type of arrival to ED, shock index, ICD-10 group (based on ICD-10, patients were grouped into gastrointestinal bleeding, obstetrics/gynecology (OBGYN) bleeding, trauma, anemia, cancer-related anemia, hematological disorders and other), use of anticoagulants, time from ED arrival to transfusion in minutes, blood product type administered (uncrossmatched RBC, fresh frozen plasma, platelets, coagulation factor concentrate), use of other medication (calcium, tranexamic acid, fluids, vasopressors), laboratory studies (pre- and post-transfusion hemoglobin and platelet counts), disposition from the ED, length of stay in the hospital in days, hemorrhage control interventions performed within the first 24 h after arrival at the ED (endoscopy, interventional radiology, surgery, pelvic binder, tourniquet) and in-hospital mortality.

The shock index was calculated as heart rate divided by systolic blood pressure, using the first vital signs recorded in the ED. Where more than one hemoglobin measurement was available before transfusion, the value closest in time to the start of transfusion was used. Each patient was assigned a single primary indication, determined from the treating clinician’s documentation.

Statistical analysis: Descriptive analysis was performed using IBM SPSS Statistics version 31 (IBM Corp., Armonk, NY, USA).

Qualitative data are presented as absolute numbers (*n*) and percentages (%). Quantitative data are presented as medians with interquartile ranges (IQRs; Q1–Q3). The Shapiro–Wilk test was used to assess normality. Non-normally distributed continuous variables were compared using the Mann–Whitney U test. Between-group differences for these variables were estimated using the independent-samples Hodges–Lehmann estimator and are presented with 95% confidence intervals (CIs). Categorical variables were compared using Pearson’s chi-square test or Fisher’s exact test when expected cell counts were small. For 2 × 2 contingency tables, unadjusted odds ratios (ORs) with 95% CIs were calculated. ORs were not estimated when a zero cell precluded a stable conventional estimate. All tests were two-sided, and statistical significance was set at *p* < 0.05. Analyses were based on available data; denominators are reported where they differed from the total cohort.

Use of artificial intelligence: During the preparation of this manuscript, the authors used Claude (Anthropic, San Francisco, CA, USA) to translate draft text from Lithuanian into English. The tool was not used for data extraction, statistical analysis, or clinical interpretation. All output was reviewed and edited by the authors, who take full responsibility for the content of this publication.

## 3. Results

During the study period, 670 patients met the inclusion criteria: 367 at the Lithuanian academic center and 303 across the two Polish academic centers. Patient characteristics and primary clinical indication categories are presented in Table 1.

GI bleeding was significantly more common in the Lithuanian center cohort (48.8% vs. 22.8%; OR 3.22 [95% CI 2.30–4.53]; *p* < 0.001), whereas anemia and malignancy-related anemia were more frequent in the Polish center cohort (anemia: 34.7% vs. 18.3%, OR 0.42 [95% CI 0.29–0.6]; *p* < 0.001; cancer: 26.4% vs. 7.6%, OR 0.23 [95% CI 0.15–0.37]; *p* < 0.001) (Table 1). Trauma presentations were more frequent in the Lithuanian center cohort (11.4% vs. 3.3%; OR 3.79 [95% CI 1.87–7.68]; *p* < 0.001). Anticoagulant use was significantly higher in the Lithuanian center cohort (21.3% vs. 12.8%; OR 1.84 [95% CI 0.21–2.81]; *p* = 0.004). These comparisons describe differences in the distribution of documented primary indication categories among transfused patients.

The median shock index was significantly higher in the Lithuanian center cohort than in the Polish center cohort (0.9; [IQR 0.7–1.2] vs. 0.7; IQR 0.6–0.9; 0.2 [95% CI 0.1–0.3]; *p* < 0.001). Emergency-release uncrossmatched RBC units were more frequently administered in the Lithuanian center cohort (133 [36.9%] vs. 47 [15.6%]; OR 3.18 [95% CI 2.18–4.64]; *p* < 0.001), as was fresh frozen plasma (71 [19.5%] vs. 3 [1%]; OR 24.23 [95% CI 7.55–77.8]; *p* < 0.001). Conversely, platelet transfusions were more common in the Polish center cohort (17 [4.6%] vs. 45 [14.9%], OR 0.28 [95% CI 0.16–0.5]; *p* < 0.001). The median time to transfusion was longer in the Lithuanian center cohort than in the Polish center cohort (150 [IQR 75–256] vs. 117 [IQR 103–154], 23 [95% CI 9–39]; *p* = 0.002).

Other medication comparisons are shown in Table 2.

Tranexamic acid, calcium, and vasopressors were documented more frequently in the Lithuanian center cohort, whereas the recorded volume of intravenous fluids was similar between the cohorts (Table 2).

Pre-transfusion hemoglobin use was substantially lower in the Lithuanian center cohort than in the Polish center cohort (64.5 g/L [IQR 50–86] vs. 119 g/L [IQR 83–139]; the Hodges–Lehmann difference was −43 g/L [95% CI −49 to −38]; p<0.001). In contrast, pre-transfusion platelet counts were broadly similar between the cohorts (210 × $10^{9}$/L [IQR 132–289] vs. 225 × $10^{9}$/L [IQR 261–298]; the Hodges–Lehmann difference was −12 × $10^{9}$/L [95% CI −32 to 5]; p=0.173).

Hemorrhage control interventions performed within the first 24 h after arrival at the EDs are shown in Table 3. For patients who underwent these procedures, the median time to intervention differed between the cohorts. In the Lithuanian center cohort, the median time to interventional radiology was 3.75 h (IQR 2.3–5.25). For endoscopy, the median time to the procedure was 2.5 h (IQR 2–3.88) in the Lithuanian center cohort versus 5 h (IQR 1.6–20.75) in the Polish center cohort (Hodges–Lehmann difference −2.5 [95% CI −6.5–0]; *p* = 0.066). The median time to surgery was 1.5 h (IQR 1–3) in the Lithuanian center cohort and 1.5 h (IQR 0.5–5)) in the Polish center cohort (Hodges–Lehmann difference 0 [95% CI −2–1.5]; *p* = 0.746).

Hospital admission was significantly more frequent in the Lithuanian cohort than in the Polish cohort (350 (95.4%) vs. 112 (37.0%); OR 0.03 [95% CI 0.02–0.05]; *p* < 0.001). Among admitted patients, the median hospital length of stay was significantly longer in the Lithuanian cohort compared with the Polish cohort (6 (IQR 3–11) vs. 1 (IQR 1–5); [95% CI 3–4]; *p* < 0.001). In-hospital mortality was 33.5% (*n* = 123) in the Lithuanian cohort and 13.9% (*n* = 42) in the Polish cohort (OR 3.13 [95% CI 2.12–4.63]; *p* < 0.001).

## 4. Discussion

In this retrospective comparison of one Lithuanian and two Polish academic EDs, transfused patient populations, documented product use, transfusion timing, adjunctive therapies, and unadjusted hospital outcomes differed between the participating center cohorts. Age and sex distributions were similar, and both cohorts consisted predominantly of older adults, consistently with previous ED transfusion studies [12,16]. GI bleeding, trauma, anticoagulant use, higher shock index values, and lower pre-transfusion hemoglobin were more common in the Lithuanian center cohort. In contrast, anemia and malignancy-related anemia were more frequent in the Polish center cohort.

The observed differences should be interpreted as differences in the characteristics and documented management of transfused patients at the participating centers. The Lithuanian center cohort had more frequent administration of emergency-release RBC units, fresh frozen plasma, tranexamic acid, calcium, and vasopressors, whereas platelet transfusion was more frequent in the Polish center cohort. These associations may be influenced by the case mix, referral pathways, blood bank logistics, emergency-release policies, local treatment pathways, and documentation practices. Because this study did not systematically collect a clinician-recorded indication or rationale for each component, measures of active bleeding severity, tissue hypoxia, or protocol adherence, it cannot determine why individual transfusions were initiated, whether they were appropriate, or whether one observed practice was preferable to another.

Pre-transfusion hemoglobin was substantially lower in the Lithuanian center cohort than in the Polish center cohort. In the context of the observed differences in primary indication categories and shock index values, this finding suggests that the cohorts represented clinically different transfused populations. It does not, however, establish a hemoglobin threshold for transfusion or demonstrate the relative contribution of symptoms, hemodynamic status, active bleeding, or other clinical factors to individual transfusion decisions. Current transfusion guidance recommends interpreting hemoglobin in the context of the overall clinical scenario, but this principle is presented here as external clinical guidance rather than as a conclusion demonstrated by our dataset [2,5,6,8].

Admission rates, hospital length of stay, and in-hospital mortality differed between the cohorts. These were unadjusted comparisons and should not be interpreted as effects of transfusion practice, product selection, or time to transfusion. The cohorts differed in several measured variables, while potentially important factors—including comorbidity burden, final diagnosis, active bleeding severity, definitive interventions, illness severity, treatment limitations, and local admission pathways—were not controlled for. The outcome results therefore describe the clinical trajectories of the transfused populations at the participating centers, rather than the comparative effectiveness of ED transfusion strategies. Recent observational studies have examined associations between the timing and location of RBC transfusion and outcomes in GI bleeding; however, differences in populations, clinical settings, and study designs limit direct comparison with the present descriptive cohort [20,21].

The rates of hemorrhage-control intervention mirrored the difference in the case mix. Endoscopy, interventional radiology and surgery within 24 h of ED arrival were all substantially more frequent in the Lithuanian center cohort, consistent with the predominance of acute gastrointestinal bleeding and trauma at that site, whereas the Polish center cohort was weighted towards anemia and malignancy, where transfusion itself is often the definitive intervention. This is reinforced by the disposition data: only 37% of Polish center patients were admitted, so the majority were never candidates for an inpatient hemostatic procedure within the study window. No Polish center patient underwent interventional radiology within 24 h, which is consistent with a cohort in which active arterial bleeding was uncommon. Timing data are more informative than rates in this respect. The median time to surgery was identical in the two cohorts (1.5 h), suggesting that when definitive surgical hemostasis was judged necessary, both systems prioritized it comparably. The time to endoscopy was shorter in the Lithuanian center cohort (2.5 vs. 5 h), but this comparison did not reach significance and rests on only 13 Polish center cohort procedures. The wide Polish center cohort interquartile range, extending beyond 20 h, indicates that endoscopy in that cohort spanned urgent and semi-elective indications, whereas the narrow Lithuanian center cohort range (2–3.88 h) is characteristic of resuscitative endoscopy for active bleeding. Both cohorts nevertheless fell within the 24 h window recommended for upper gastrointestinal bleeding. Taken together with the transfusion timing data, these findings suggest that the two systems differed in which patients required hemostatic intervention, rather than in how urgently intervention was delivered once indicated [5,20,21,27].

This comparative study provides insights relevant to healthcare system development and transfusion service organization in Central and Eastern Europe. Both the Lithuanian and Polish centers’ systems demonstrated functional capacity for rapid ED transfusions, appropriate use of component therapy, and access to hemostatic interventions. However, the substantially higher admission rate in the Lithuanian center cohort (95.4% vs. 37%) warrants consideration: this difference likely reflects both the case mix (higher acuity in the Lithuanian center cohort) and possible institutional policies regarding inpatient admission. Whether the lower admission rate in the Poland center cohort reflects premature discharge of high-risk patients or appropriate recognition that transfusion alone (without other interventions) can safely stabilize patients for outpatient management remains unclear from this retrospective dataset. Future prospective studies with standardized outcome tracking (72 h return visits, mortality at 30 days, re-transfusion rates) would clarify whether outcome differences are case-mix-related or reflect true differences in discharge safety protocols.

This study has several limitations. First, its retrospective design relies on routinely documented data and does not support causal inference. Second, it included only one Lithuanian and two Polish academic EDs; consequently, findings should not be generalized to national transfusion practice in Lithuania or Poland. Third, the cohorts may differ because of center-specific case mixes, referral pathways, availability of outpatient care, blood bank procedures, emergency-release policies, admission practices, coding, and documentation. Fourth, prospectively recorded clinical rationales for transfusion and measures of active bleeding severity, physiologic tissue hypoxia, comorbidity burden, definitive hemorrhage control, and protocol adherence were not available; therefore, we could not assess the appropriateness of transfusions or identify their clinical drivers. Fifth, post-transfusion laboratory data were substantially and differentially missing, particularly in the Polish cohort, preventing a reliable evaluation of laboratory response or transfusion effectiveness. Sixth, outcomes were compared without adjustment for baseline differences and unmeasured confounding, so observed differences in admission, length of stay, and mortality should be interpreted descriptively. Finally, patients were assigned a single primary indication category despite the possibility of overlapping conditions contributing to transfusion.

## 5. Conclusions

This retrospective comparison of transfused patients in one Lithuanian and two Polish academic EDs found substantial differences in case mixes, blood product use, transfusion timing, and unadjusted hospital outcomes. The Lithuanian center cohort had a higher proportion of gastrointestinal bleeding and trauma, lower pre-transfusion hemoglobin, and a higher median shock index, while anemia- and malignancy-related presentations and platelet transfusions were more frequent in the Polish center cohort. These results describe the participating centers and should not be generalized to national practice or interpreted as evidence of causal differences in transfusion decision-making, appropriateness, or outcomes. Prospective studies with standardized data collection, site-level analyses, documented indications, and adjustment for illness severity and clinical pathways are needed.

## Figures and Tables

**Table 1:** Patient characteristics.

Variable	Lithuanian Center, *n* = 367	Polish Centers, *n* = 303	OR (95% CI); *p*-Value
Age (years (IQR))	68 (54–81)	71 (58–81)	−2.0 (95% CI −5.0–1.0); *p* = 0.149
Gender (male *n* (%))	197 (53.7)	152 (50.2)	OR 0.87 (95% CI 0.64–1.18); *p* = 0.365
Anticoagulant use, *n* (%)	78 (21.3)	38 (12.8)	OR 1.84 (95% CI 0.21–2.81); *p* = 0.004
GI bleeding, *n* (%)	179 (48.8)	69 (22.8)	OR 3.22 (95% CI 2.30–4.53); *p* < 0.001
Anemia, *n* (%)	67 (18.3)	105 (34.7)	OR 0.42 (95% CI 0.29–0.6); *p* < 0.001
Cancer, *n* (%)	28 (7.6)	80 (26.4)	OR 0.23 (95% CI 0.15–0.37); *p* < 0.001
Trauma, *n* (%)	42 (11.4)	10 (3.3)	OR 3.79 (95% CI 1.87–7.68); *p* < 0.001
Hematological disorder, *n* (%)	5 (1.4)	11 (3.6)	OR 0.37 (95% CI 0.17–1.07); *p* = 0.056
Obstetric/gynecologic bleeding, *n* (%)	5 (1.4)	6 (2)	OR 0.68 (95% CI 0.21–2.26); *p* = 0.557
Anticoagulant overdose, *n* (%)	4 (1.1)	0	*p* = 0.131
Other, *n* (%)	4 (1.1)	0	*p* = 0.131

**Table 2:** Other medications used in transfused patients.

Medication	Lithuanian Center, *n* = 367	Polish Centers, *n* = 303	OR [95% CI]; *p*-Value
Tranexamic acid, *n* (%)	71 (19.3)	24 (7.9)	2.78 [1.7–4.54]; *p* < 0.001
Calcium, *n* (%)	78 (21.4)	9 (3)	8.85 [4.36–17.98]; *p* < 0.001
Vasopressors, *n* (%)	64 (17.5)	11 (3.6)	5.64 [2.92–10.92]; *p* < 0.001
Intravenous fluids, mL, median (IQR)	500 (500–1000)	500 (500–1000)	0 [0-0]; 0.952

**Table 3:** Hemorrhage control interventions performed within the first 24 h after arrival at the EDs.

Variable	Lithuanian Center, *n* = 367	Polish Centers, *n* = 303	OR [95% CI]; *p*-Value
Endoscopy, *n* (%)	128 (34.9)	13 (4.3)	11.95 [6.59–21.68]; *p* < 0.001
Interventional radiology, *n* (%)	50 (13.6)	0	<0.001
Surgery, *n* (%)	35 (9.5)	7 (2.3)	4.46 [1.95–10.19] <0.001
Tourniquet, *n* (%)	1 (0.3)	0	1
Pelvic binder, *n* (%)	1 (0.3)	0	1

## Data Availability

The original contributions presented in this study are included in the article. Further inquiries can be directed to the corresponding authors.

## Abbreviations

The following abbreviations are used in this manuscript:

ED	Emergency department
RBC	Red blood cell
PBM	Patient blood management
HB	Hemoglobin
IQR	Interquartile range
OR	Odds ratio
CI	Confidence interval
